# Aristolochic acid, a plant extract used in the treatment of pain and linked to Balkan endemic nephropathy, is a regulator of K2P channels

**DOI:** 10.1111/bph.13465

**Published:** 2016-04-05

**Authors:** Emma L Veale, Alistair Mathie

**Affiliations:** ^1^ Medway School of PharmacyUniversity of KentKentUK

## Abstract

**Background and Purpose:**

Aristolochic acid (AristA) is found in plants used in traditional medicines to treat pain. We investigated the action of AristA on TREK and TRESK, potassium (K2P) channels, which are potential therapeutic targets in pain. Balkan endemic nephropathy (BEN) is a renal disease associated with AristA consumption. A mutation of TASK‐2 (K_2P_5.1) channels (T108P) is seen in some patients susceptible to BEN, so we investigated how both this mutation and AristA affected TASK‐2 channels.

**Experimental Approach:**

Currents through wild‐type and mutated human K2P channels expressed in tsA201 cells were measured using whole‐cell patch‐clamp recordings in the presence and absence of AristA.

**Key Results:**

TREK‐1‐ and TREK‐2‐mediated currents were enhanced by AristA (100 μM), whereas TRESK was inhibited. Inhibition of TRESK did not depend on the phosphorylation of key intracellular serines but was completely blocked by mutation of bulky residues in the inner pore (F145A_F352A). The TASK‐2_T108P mutation markedly reduced both current density and ion selectivity. A related mutation (T108C) had similar but less marked effects. External alkalization and application of flufenamic acid enhanced TASK‐2 and TASK‐2_T108C current but did not affect TASK‐2_T108P current. AristA (300 μM) produced a modest enhancement of TASK‐2 current.

**Conclusions and Implications:**

Enhancement of TREK‐1 and TREK‐2 and inhibition of TRESK by AristA may contribute to therapeutically useful effects of this compound in pain. Whilst AristA is unlikely to interact directly with TASK‐2 channels in BEN, loss of functional TASK‐2 channels may indirectly increase susceptibility to AristA toxicity.

**Abbreviations**
AristaA: aristolochic acid
BEN: Balkan endemic nephropathy
FFA: flufenamic acid

## Tables of Links

**Table:** 

**TARGETS**	
TASK‐2 (K_2P_5.1) channel	TREK‐1 (K_2P_2.1) channel
TASK‐3 (K_2P_9.1) channel	TREK‐2 (K_2P_10.1) channel
THIK‐1 (K_2P_13.1) channel	TRESK (K_2P_18.1) channel
TRAAK (K_2P_4.1) channel	TRPA1 channel

**LIGANDS**
Flufenamic acid (FFA)

These Tables list key protein targets and ligands in this article which are hyperlinked to corresponding entries in http://www.guidetopharmacology.org, the common portal for data from the IUPHAR/BPS Guide to PHARMACOLOGY (Pawson *et al.*, 2014) and are permanently archived in the Concise Guide to PHARMACOLOGY 2015/16 (Alexander *et al.*, 2015).

## Introduction

Aristolochic acid (AristA) is found in plants of the *Aristolochiaceae* family, which have been used widely in traditional medicine for thousands of years. These plants are mentioned in early first‐century Roman texts as components of frequently ingested medicines to treat a variety of conditions including asthma, hiccups, spasms, pains and expulsion of afterbirth, and they were described as components of certain Chinese herbal medicines in the fifth century AD (Scarborough, 2011). Although these herbal remedies were used for a variety of conditions, a recurring theme was their use in many cultures for the treatment of pain. For example, *Aristolochia clematitis* (birthwort) has been used globally in childbirth, chronic pain and arthritis (Debelle *et al.*, 2008), and Asarum caudatum (wild ginger) has been used in Chinese and Native American herbal remedies for migraine, intestinal pain and knee pain (Smith, 1929). The use of extracts of this family of plants in the treatment of pain was particularly widespread in Chinese herbal medicine. *Aristolochia debilis* has been used for headache, abdominal pain and epigastric pain, *Aristolochia manshuriensis*, as part of complex prescriptions, as an anti‐inflammatory treatment and *Aristolochia fangchi*, also in complex prescriptions, as an analgesic (IARC, 2002, 2012).

AristA has, however, been associated with nephropathy leading to end‐stage renal disease and also with urological malignancies. This first came to light when cases of nephritis and kidney failure were seen in a group of women in Belgium who had all taken a particular weight‐loss supplement, which, inadvertently, contained AristA (Vanherweghem *et al.*, 1993; Debelle *et al.*, 2008). A recent pair of studies identified an AristA mutational signature in upper urinary tract cancer patients from Taiwan (Hoang *et al.*, 2013; Poon *et al.*, 2013) and showed that this carcinogenic effect of AristA is amongst the most potent yet described. In animal studies, either brief exposure to high concentrations (e.g. 50 mg·kg^−1^ for 3 days) or extended exposure to low concentrations (e.g. 0.1 mg·kg^−1^ for 12 months) causes a variety of malignant tumours (IARC, 2012). As a result, AristA is now banned as a component of herbal medicines in many countries, including the UK and USA (Debelle *et al*., 2008). Despite this, its use is still widespread (Debelle *et al.*, 2008). For example, fruits from a variety of plants of the *Aristolochiaceae* family are freely available in Iran and used to treat headache and back pain (Ardalan *et al.*, 2015).

Balkan endemic nephropathy (BEN) is a renal disease restricted to rural areas of the Balkans and characterized by insidious onset, chronic renal failure and strong association with urothelial carcinoma of the upper urinary tract (Grollman *et al.*, 2007). It is widely believed that BEN is caused by low‐level AristA exposure, probably from contamination of wheat flour seeds by A. clematitis (Grollman *et al.*, 2007; De Broe, 2012; Bui‐Klimke and Wu, 2014). Because, like the diseases associated with AristA‐containing herbal medicines, BEN is linked to AristA, this has prompted the use of the term aristolochic acid nephropathy to describe all related renal diseases associated with consumption of this compound (e.g. De Broe, 2012). It is of interest that a number of patients predisposed to BEN have been shown to carry a mutation of the physiological important renal (Sepúlveda *et al.*, 2015) two‐pore‐domain potassium (K2P) channel, TASK‐2 (K_2P_5.1) (Toncheva *et al.*, 2014).

Despite the profound toxicity issues associated with AristA, we were intrigued by the widespread use of this compound in traditional medicine in the treatment of pain. A role for K2P channels as therapeutic targets for the treatment of pain has been proposed (e.g. Alloui *et al.*, 2006; Mathie, 2010; Devilliers *et al.*, 2013; Mathie and Veale, 2015), in particular the TREK (K_2P_2.1, K_2P_4.1, K_2P_10.1) and TRESK (K_2P_18.1) families of K2P channels (as reviewed by Mathie and Veale, 2015). The first aim of this study, therefore, was to investigate the action of AristA on these K2P channels to determine whether this may contribute to its apparent therapeutic usefulness in the treatment of pain. The second aim of this study was to describe how the mutation of TASK‐2 channels seen in patients susceptible to BEN influenced the functional properties of TASK‐2 channels and to determine whether AristA altered the activity of this K2P channel and thus whether this may contribute to the putative action of AristA in BEN.

## Methods

### Group sizes

The exact group size (*n*) for each experimental group/condition is provided, and *n* refers to independent values, not replicates. Data subjected to statistical analysis have an *n* of at least five per group.

### Randomization

When comparisons are made between different recording conditions or different, mutated, forms of a channel, recordings were alternated between one condition and the other on a given experimental day.

### Blinding

No blinding was undertaken in this study. It is not a usual procedure for this form of study and cannot be applied retrospectively.

### Normalization

No normalization of primary data was performed.

### Statistical comparison

Group mean values and statistical analysis use independent values. When comparing groups, a level of probability (*P* < 0.05) was deemed to constitute the threshold for statistical significance.

### Cell culture

tsA201 cells (ECACC; Sigma‐Aldrich, Gillingham, Dorset, UK), modified human embryonic kidney 293 cells, were grown in a monolayer tissue culture flask maintained in a growth medium, which was composed of 88% minimum essential media with Earle's salts and 2 mM l‐glutamine, 10% of heat‐inactivated fetal bovine serum, 1% penicillin (10 000 U·mL^−1^) and streptomycin (10 mg·mL^−1^) and 1% non‐essential amino acids. The cells were placed in an incubator at 37°C with a humidified atmosphere of 95% oxygen and 5% carbon dioxide. After 2 or 3 days, when the cells were 70 to 90% confluent, they were split and resuspended in a four‐well plate containing 13‐mm‐diameter cover slips (poly‐d‐lysine coated) in 0.5 mL of media, ready to be transfected the next day.

### Transfection

For the electrophysiological experiments, the pcDNA3.1 vector was cloned with the gene of interest [hTREK‐1 (or K_2P_2.1, as designated by Alexander *et al.*, 2015), hTREK‐2 (K_2P_10.1), hTRAAK (K_2P_4.1), hTRESK (K_2P_18.1), hTASK‐2 (K_2P_5.1) and hTASK‐3 (K_2P_9.1) wild type (WT) and mutated]. The hTHIK‐1 (K_2P_13.1) gene was in the vector pcDNA3.1_NEO_N8. These vectors and a similar vector containing GFP were incorporated into the tsA201 cells (0.5 μg per well) using the calcium phosphate method. The cells were incubated for 6–12 h at 37°C in 95% oxygen and 5% carbon dioxide. Cells were then washed using a PBS solution, and new media added to each well. The cells were used for experiments after 24 h.

### Mutations

Point mutations were introduced by site‐directed mutagenesis into the K2P channel clones using the Quikchange kit (Stratagene, Amsterdam, the Netherlands). A pair of short (25–35 bases) complementary oligonucleotide primers, incorporating the intended mutation, were synthesized (Eurofins MWG Operon, Ebersberg, Germany). Mutant DNA constructs were sequenced (Eurofins MWG Operon) to confirm the introduction of the correct mutated bases.

### Whole‐cell patch‐clamp electrophysiology

Currents were recorded using the whole‐cell patch clamp in a voltage clamp configuration in tsA201 cells transiently transfected with the channel of interest. The cover slip with the cells was placed in a recording chamber filled with an external medium composed of 145 mM NaCl, 2.5 mM KCl, 3 mM MgCl_2_, 1 mM CaCl_2_ and 10 mM HEPES (pH to 7.4, using NaOH). In experiments where external [K] was varied, NaCl was replaced with KCl to give the appropriate concentration of K. The internal medium used in the glass pipette comprised 150 mM KCl, 3 mM MgCl_2_, 5 mM (or 0.1 mM) EGTA and 10 mM HEPES (pH to 7.4, using KOH). Modulatory compounds were applied by bath perfusion at a rate of 4–5 mL·min^−1^. Complete exchange of bath solution usually occurred within 100–120 s; as such, any effect of these compounds on current amplitude was only measured once the response had reached steady state.

All the data presented have been collected at room temperature (19–22°C). The transfected cells were detected using a fluorescent microscope with UV light. Cells were voltage clamped using an Axopatch 1D or Axopatch 200B amplifier (Molecular Devices, Sunnyvale, CA, USA) and low‐pass filtered at 5 kHz before sampling (2–10 kHz) and online capture.

In order to study the potassium leak current, a ‘step‐ramp’ voltage protocol was used. For the step component of the protocol, cells were hyperpolarized from a holding voltage of −60 to −80 mV for 100 ms then stepped to −40 mV for 500 ms. For the ramp, cells were then stepped to −120 mV for 100 ms, followed by a 500 ms voltage ramp to +20 mV and a step back to −80 mV for another 100 ms, before being returned to the holding voltage of −60 mV. This protocol was composed of sweeps that lasted 1.5 s (including sampling at the holding voltage) and was repeated once every 5 s (see also Veale *et al.*, 2014b).

### Data analysis

For analysis of outward current, we measured the current difference between the −80 and −40 mV steps. The current–voltage graphs were obtained from the ramp change in voltage between −120 and +20 mV. The currents obtained with the imposed voltage protocol were recorded and analysed using pclamp 10.2 software and Microsoft Excel. For each cell, the current amplitude (pA) was normalized to the cell capacitance (pF).

Data are expressed as mean ± SEM, and *n* represents the number of cells used for the experiment. The statistical analyses used either Student's *t*‐test (both paired and unpaired) or a one‐way ANOVA with *post hoc* Dunnett's multiple comparisons test, using graphpad prism 6.02 (GraphPad Software, Inc., La Jolla, CA, USA). For the *t*‐test, the differences between means were considered as significant for *P* < 0.05. For the Dunnett's test, data were considered significantly different at the <0.05 level (confidence interval > 95% for the difference between the two compared means). Concentration–response data were fitted using the Hill equation. The data and statistical analysis comply with the recommendations on experimental design and analysis in pharmacology (Curtis *et al.*, 2015).

### Chemicals

**Figure 1:**
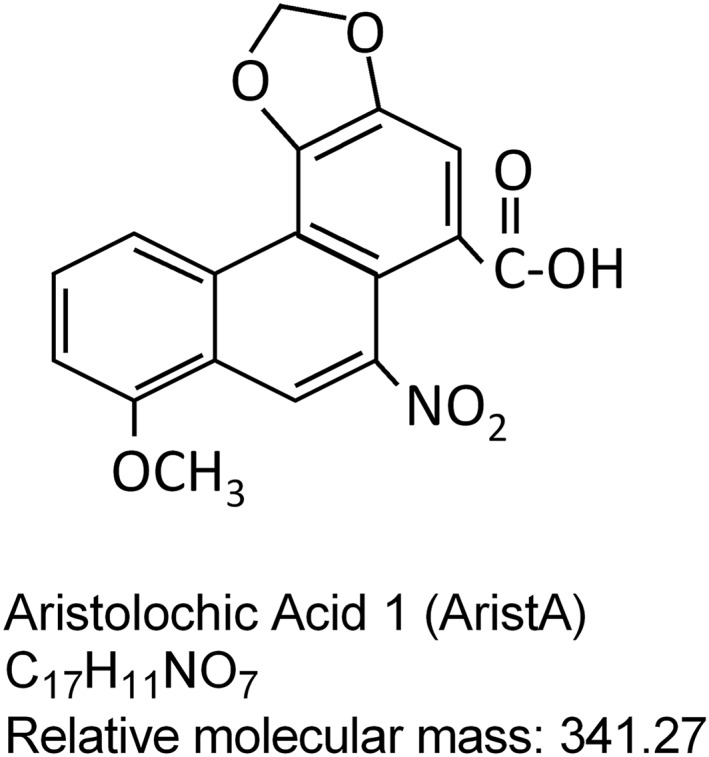
Structure of AristA. Adapted from IARC (2012).

All fine chemicals were purchased from Sigma‐Aldrich. Flufenamic acid (FFA) stock (10 mM) was made up in ethanol and diluted fresh in external solution before use (pH adjusted to 7.4). Aristolochic acid I (AristA) stock (50 mM) was made up in water and diluted in fresh external before use (pH adjusted to 7.4). Extracts of *Aristolochia* species comprise, primarily, of a mixture of aristolochic acid I and its demethoxylated derivative, aristolochic acid II. In this study, we used purified aristolochic acid 1 (Figure 1), and the term aristolochic acid (AristA) is used to denote this.

## Results

### AristA enhances TREK‐1 and TREK‐2 channel currents

**Figure 2:**
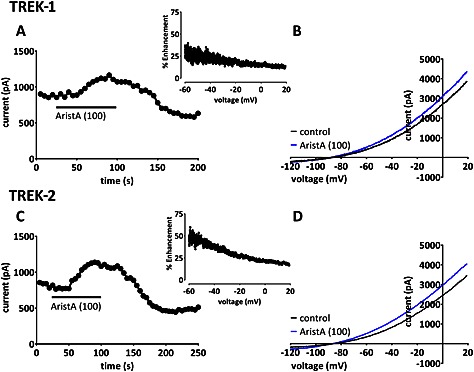
Aristolochic acid (AristA) enhances TREK‐1 and TREK‐2 currents, with apparent voltage‐dependence. (A) Time course plot showing an enhancement of human TREK‐1 current transiently expressed in tsA201 cells by AristA (100 μM). Each point is a 5 ms average of the difference current between that at −40 mV and that at −80 mV (see Methods for detailed description of voltage ramp protocol). (B) TREK‐1 currents evoked by ramp changes in voltage from −120 to +20 mV in control conditions and in the presence of 100 μM AristA. Inset shows percentage enhancement of TREK‐1 current by 100 μM AristA plotted as a function of voltage. (C) Time course plot showing an enhancement of human TREK‐2 current by AristA (100 μM). Inset shows percentage enhancement of TREK‐2 current by 100 μM AristA plotted as a function of voltage. (D) TREK‐2 currents evoked by ramp changes in voltage in control conditions and in the presence of 100 μM AristA.

Application of AristA (aristolochic acid I, see Section on Methods; 100 μM) resulted in an enhancement of current through TREK‐1 and TREK‐2 channels of 26 ± 6% (mean ± SEM, *n* = 6) and 44 ± 11% (*n* = 6), respectively, when current is measured as the difference current between that seen at −40 mV and that at −80 mV (see Section on Methods). Enhancement of both currents was rapid and easily reversible (Figure 2A–D). There was some voltage‐dependence to the enhancement for both currents. This is illustrated for exemplar cells in the range −60 to +20 mV for either current (Figure 2 insets). Enhancement of TREK‐1 was 42 ± 9% (*n* = 6) at −60 mV, which was significantly larger than the 17 ± 6% (*n* = 6) enhancement at +20 mV in the same cells. For TREK‐2, enhancement was 50 ± 14% (*n* = 5) at −60 mV compared with 17 ± 3% (*n* = 5) at +20 mV in the same cells. Thus, enhancement was greater near the reversal potential of the currents and therefore near the resting membrane potential of cells in which they are expressed. In some recordings for both TREK‐1 and TREK‐2 (Figure 2A and C), there does appear to be a biphasic response to AristA (100 μM) with an ‘over‐recovery’ on washout leading to a sustained reduced current amplitude compared with that measured before application of the drug. However, a lower concentration of the compound (1 μM) had no detectable effect on either TREK‐1 or TREK‐2.

**Figure 3:**
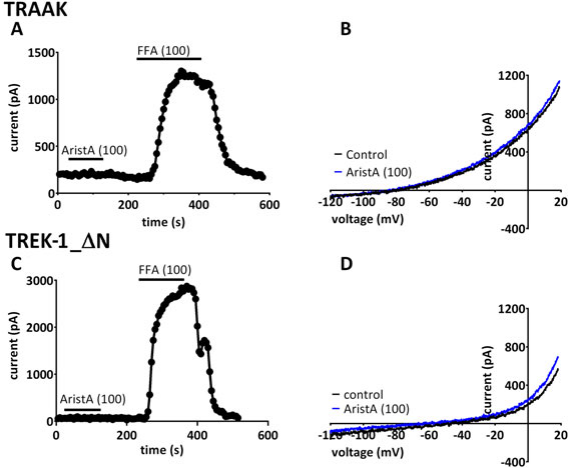
Aristolochic acid (AristA) has no effect on the closely related two‐pore‐domain potassium channel, TRAAK and the N‐terminally truncated TREK‐1 isoform (TREK‐1_ΔN). (A) Time course plot showing lack of effect of AristA (100 μM) on human TRAAK isoform 2 (the sequence of this isoform differs from the canonical sequence by the addition of 26 amino acids, preceding the start codon of isoform 1). (B) TRAAK currents evoked by ramp changes in voltage in control conditions and in the presence of 100 μM AristA. (C) Time course plot showing lack of effect of AristA (100 μM) on the alternative translational initiation isoform of TREK‐1 (TREK‐1_ΔN), where the first 41 amino acids of wild‐type TREK‐1 are missing. (D) TREK‐1_ΔN currents evoked by ramp changes in voltage in control conditions and in the presence of 100 μM AristA.

By contrast, AristA (100 μM) had little effect on currents mediated through TRAAK channels (Figure 3A and B) with an enhancement of 8 ± 5% (*n* = 5), or on the short form of TREK‐1 channels (TREK‐1_ΔN, Figure 3C and D), formed by alternative translation initiation (Thomas *et al.*, 2008; Veale *et al.*, 2010; 2014a), with an enhancement of −4 ± 5% (*n* = 5). This was despite the fact that both of these channels could be substantially enhanced by flufenamic acid (Veale *et al.*, 2014a) in recordings from the same cells (Figure 3A and C).

TREK channel activity is subject to convergent, polymodal regulation by a variety of factors including temperature, pressure and the phosphorylation state of the channel, and this may influence the response of the channels to other regulatory compounds (Bagriantsev *et al.*, 2012). Recent experiments by Bagriantsev *et al.* (2012) showed that a triple glycine (3G) mutant, which decouples the intracellular C terminal tail of the channels from the pore‐forming core, rendered the channels insensitive to polymodal regulation by the aforementioned factors. Bagriantsev *et al.* (2013) found that the TREK channel activator, ML67‐33, still activated these mutated channels. We have made the corresponding 3G mutant of TREK‐2 (TREK‐2_I318G_G319_D320G). These channels were still enhanced by 100 μM AristA (26 ± 5%, *n* = 5), and this enhancement was not significantly different to that seen for WT TREK‐2 channels (44 ± 11%, *n* = 6). This suggests that enhancement of TREK‐2 channels by AristA occurs independently from polymodal regulation through the intracellular C terminal tail of the channel.

### AristA inhibits TRESK channel currents

**Figure 4:**
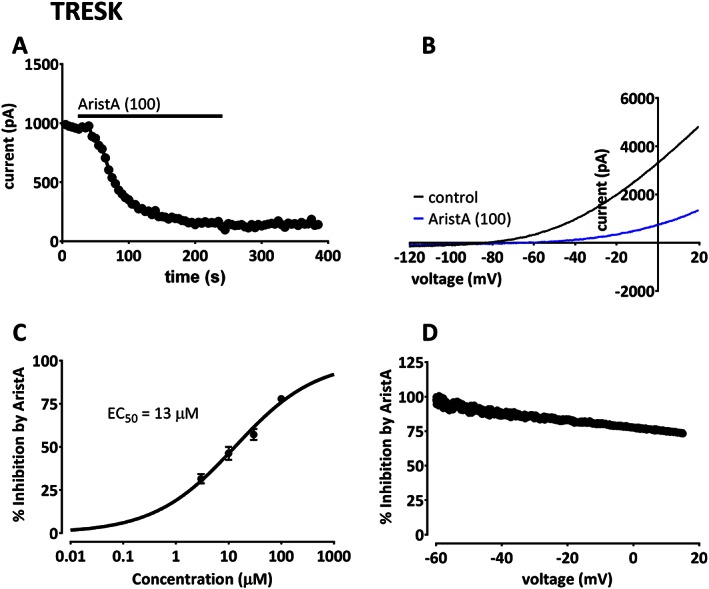
Aristolochic acid (AristA) inhibits TRESK‐mediated currents with apparent voltage‐dependence. (A) Time course plot showing an inhibition of human TRESK current by AristA (100 μM). (B) TRESK currents evoked by ramp changes in voltage in control conditions and in the presence of 100 μM AristA. (C) Concentration–response curve for AristA inhibition of TRESK current. (D) Percentage inhibition of TRESK current by 100 μM AristA plotted as a function of voltage.

In contrast to the enhancement seen at high concentrations for TREK‐1 and TREK‐2 channels, we observed that AristA was an *inhibitor* of TRESK channels (Figure 4A and B). Inhibition had a fast onset but was only slowly reversible, particularly at higher concentrations such as 100 μM (Figure 4A), with a calculated 50% effective concentration of 13 ± 2 μM for AristA on TRESK and a Hill slope of 0.56 ± 0.08 (Figure 4C). As for enhancement of TREK channels, the effect of AristA was voltage‐dependent. This is illustrated for an exemplar cell in Figure 4D. Inhibition of TRESK by 100 μM AristA was 96 ± 6% (*n* = 6) at −60 mV, which was significantly larger than the 56 ± 5% (*n* = 6) inhibition seen at +20 mV in the same cells. At +20 mV, AristA had a calculated 50% effective concentration of 86 ± 3 μM with a Hill slope of 0.34 ± 0.11. Thus, as with enhancement of TREK‐1 and TREK‐2 channels, inhibition of TRESK was greater around the reversal potential of the currents and therefore around the resting membrane potential of cells in which they are expressed.

### AristA and TRESK channel mutants

TRESK channels expressed in oocytes have been shown to be directly regulated by increases in intracellular calcium through activation of the calcium/calmodulin‐dependent phosphatase calcineurin (e.g. Czirják and Enyedi, 2010). We, therefore, carried out a number of experiments to investigate the potential role of AristA in calcium‐dependent processes. In the first set of experiments, we altered the internal calcium buffer in the recording solution between 0.1 mM (‘low’ EGTA) and 5 mM (‘high’ EGTA). This had no significant effect on inhibition by AristA (100 μM) with 73 ± 4% (*n* = 6) inhibition in low EGTA and 79 ± 2% (n = 6) inhibition in high EGTA. Thus, inhibition was unaffected by the degree of intracellular calcium buffering.

For mouse TRESK channels, Czirják and Enyedi (2010) have shown that mutation of key serine residues, located in the large intracellular loop between transmembrane domains M2 and M3, can abolish the effects of phosphatase action. We thus created two mutant human TRESK channels, S252E_S264E to mimic the phosphorylated form of the channel and S252A_S264A to mimic the dephosphorylated form of the channel.

**Figure 5:**
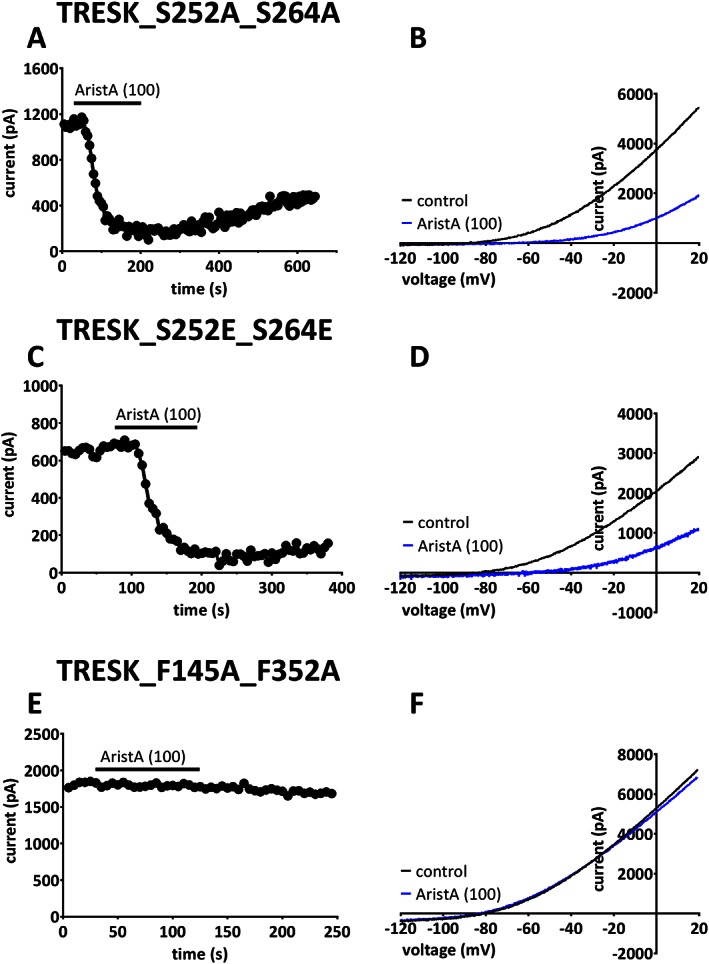
Aristolochic acid (AristA) inhibits serine phosphorylation mutants of TRESK, but its effect is significantly attenuated by the phenylalanine mutants in the inner pore. (A) Time course plot showing an inhibition of the phosphorylation mutant TRESK_S252A_S264A by AristA (100 μM). (B) TRESK_S252A_S264A currents evoked by ramp changes in voltage in control conditions and in the presence of 100 μM AristA. (C) Time course plot showing an inhibition of the phosphorylation mutant TRESK_S252E_S264E by AristA (100 μM). (D) TRESK_S252E_S264E currents evoked by ramp changes in voltage in control conditions and in the presence of 100 μM AristA. (E) Time course plot showing lack of effect on mutant TRESK_F145A_F352A by AristA (100 μM). (F) TRESK_F145A_F352A currents evoked by ramp changes in voltage in control conditions and in the presence of 100 μM AristA.

For S252A_S264A channels, current density was significantly larger than WT channels (48 ± 3 pA/pF, *n* = 31, for the mutated channels compared with 27 ± 2 pA/pF, *n* = 28, for WT TRESK channels); however, AristA (100 μM) still inhibited channel current by 80 ± 2% (*n* = 6, Figure 5A and B). For S252E_S264E channels, current density was not significantly different to WT channels (33 ± 3 pA/pF, *n* = 39). AristA (100 μM) was, again, able to inhibit current through these channels by 73 ± 5% (*n* = 5) (Figure 5C and D). The inhibition of either phosphorylation mutant by AristA was not significantly different to that of WT TRESK.

Recently, mutation of two amino acids in the M2 and M4 inner pore regions of mouse TRESK channels (equivalent to F145A_F352A in human TRESK) has been shown to occlude the action of a number of different blocking drugs of the channel, and it was proposed that this position forms a binding site for blockers targeting TRESK channels (Kim *et al.*, 2013; Bruner *et al.*, 2014). We investigated the effect of this double mutation on the action of AristA on TRESK channels. We found that AristA (100 μM) no longer inhibited current through these mutated channels (0 ± 5%, *n* = 7, Figure 5E and F).

### TASK‐2, AristA and BEN

AristA has been implicated in BEN (see Introduction). A recent paper has suggested that a number of patients suffering from BEN have a mutation in the coding region of the K2P channel, TASK‐2, namely T108P, suggesting an important role of TASK‐2 channels in BEN predisposition (Toncheva *et al.*, 2014). Given the potential importance of both AristA and TASK‐2 channels in BEN, we were interested, therefore, to determine whether AristA interacted with either (or both) of the WT or the T108P mutated form of TASK‐2. Initially, however, we characterized the properties of these T108P mutated TASK‐2 channels.

**Figure 6:**
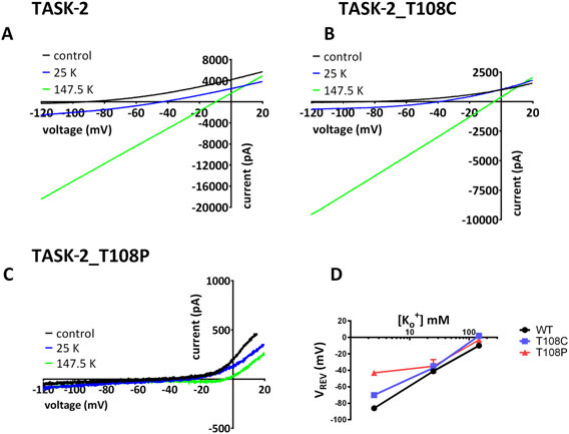
Characteristics of currents through WT TASK‐2, TASK‐2_T108C and TASK‐2_T108P channels in varying concentrations of extracellular potassium (K^+^). (A) Currents recorded through wild‐type TASK‐2 channels in 2.5 mM K^+^, 25 mM K^+^ and 147.5 mM K^+^. Currents evoked by ramp changes in voltage from −120 to +20 mV, as detailed in Methods. (B) Currents recorded through TASK‐2_T108C channels in 2.5 mM K^+^, 25 mM K^+^ and 147.5 mM K^+^. (C) Currents recorded through TASK‐2_T108P channels in 2.5 mM K^+^, 25 mM K^+^ and 147.5 mM K^+^. (D) Plot of reversal potential versus external K^+^ concentration for WT TASK‐2, TASK‐2_T108C and TASK‐2_T108P.

Figure 6A–C shows currents through WT TASK‐2 channels, TASK‐2_T108P channels and a more conservatively mutated TASK‐2 channel at the same residue, TASK‐2_T108C, in a variety of external K^+^ concentrations. In contrast to WT and TASK‐2_T108C channels, there is very little current through TASK‐2_T108P mutated channels regardless of the external K^+^ concentration. For example, in 2.5 mM external K^+^, current density for WT TASK‐2 was 52 ± 4 pA/pF (*n* = 27); for TASK‐2_T108C, it was 9 ± 1 pA/pF (*n* = 15), and for TASK‐2_T108P, it was just 2 ± 1 pA/pF (*n* = 26). Both mutations had significantly smaller current density than WT TASK‐2.

Furthermore, unlike WT TASK‐2 channels, the reversal potential of TASK‐2_T108P channels measured in different external [K^+^] are not consistent with the presence of a K^+^‐selective current (Figure 6D). For example, in 2.5 mM external K^+^, reversal of current for WT TASK‐2 was −86 ± 1 mV (*n* = 27), but for TASK‐2_T108P, this was −43 ± 3 mV (*n* = 26). It is of interest that the TASK‐2_T108C channel also showed a reduced K^+^ selectivity with a reversal potential in 2.5 mM external K^+^ of −70 ± 3 mV (*n* = 15).

### Activators of TASK‐2 channels

**Figure 7:**
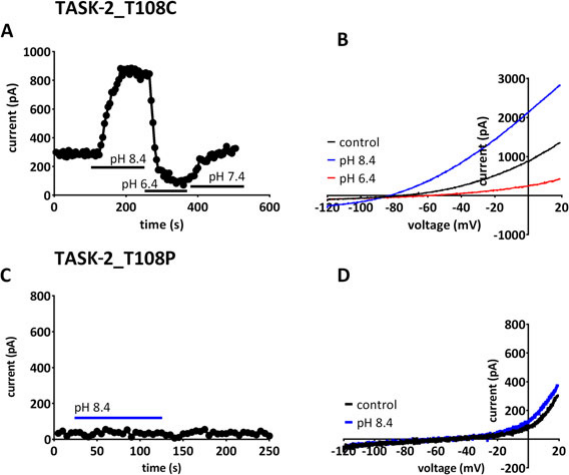
Effect of changes in extracellular pH on mutant TASK‐2 channels. (A) Representative time course for enhancement and inhibition of outward current by extracellular alkalization and acidification, respectively, through TASK‐2_T108C channels. (B) TASK‐2_T108C currents evoked by ramp changes in voltage in control solution (pH 7.4), pH 8.4 and pH 6.4. (C) Representative time course for changes of outward current by extracellular alkalization through TASK‐2_T108P channels. (D) TASK‐2_T108P currents evoked by ramp changes in voltage in control solution (pH 7.4) and in alkaline solution (pH 8.4).

We next considered whether activators of TASK‐2 channels might induce current through T108P mutated channels, as has been seen previously for other non‐functional or reduced‐function, mutated K2P channels (Ma *et al.*, 2013; Veale *et al.*, 2014a, 2014b). Changes in external pH are known to regulate TASK‐2 channels (Reyes *et al.*, 1998). Figure 7A and B illustrates that very similar regulation by external pH for TASK‐2_T108C channels to that known for WT TASK‐2 channels occurs, with clear enhancement of current at pH 8.4 and inhibition at pH 6.4 compared with pH 7.4. We found a 120 ± 17% (*n* = 9) enhancement at pH 8.4 for WT TASK‐2 and a 143 ± 22% (*n* = 9) enhancement for TASK‐2_T108C channels. The reversal potential at pH 8.4 (in 2.5 mM external K^+^) was −91 ± 3 mV (*n* = 5) for WT TASK‐2 and −76 ± 3 mV (*n* = 7) for TASK‐2_T108C channels. However, in contrast, pH 8.4 had no effect on TASK‐2_T108P channels (Figure 7C and D) with 10 ± 12% (*n* = 7) enhancement of current and a reversal potential of −52 ± 5 mV (*n* = 7) at pH 8.4.

**Figure 8:**
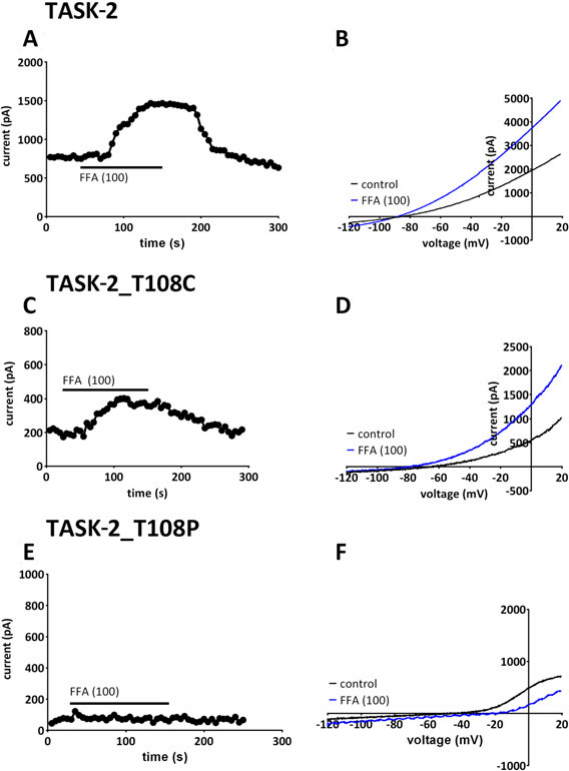
Regulation of WT TASK‐2, TASK‐2_T108C and TASK‐2_T108P mutant channels by flufenamic acid (FFA). (A) Representative time course for enhancement of outward current by FFA (100 μM) for current through WT TASK‐2 channels. (B) Current–voltage relationships for WT TASK‐2 channels in the absence and presence of FFA (100 μM). (C) Representative time course for enhancement of outward current by FFA (100 μM) for current through TASK‐2_T108C mutant channels. (D) Current–voltage relationships for TASK‐2_T108C mutant channels in the absence and presence of FFA (100 μM). (E) Representative time course demonstrating lack of effect on the outward current by FFA (100 μM) on current through TASK‐2_T108P mutant channels. (F) Current–voltage relationships for TASK‐2_T108P mutant channels in the absence and presence of FFA (100 μM).

FFA (100 μM) is a known enhancer of several K2P channels (Veale *et al.*, 2014a, 2014b; see also Figure 2). We showed that this compound is also able to enhance current through both WT TASK‐2 channels (Figure 8A and B) and TASK‐2_T108C channels (Figure 8C and D). We found an 80 ± 31% (*n* = 7) enhancement for WT TASK‐2 by FFA and a 97 ± 25% (*n* = 6) enhancement for TASK‐2_T108C channels. The reversal potential in FFA (in 2.5 mM external K) was −90 ± 3 mV (*n* = 7) for WT TASK‐2 and −72 ± 7 mV (*n* = 6) for TASK‐2_T108C channels. However, FFA (100 μM) produced no enhancement of TASK‐2_T108P channels (Figure 8E and F) with 15 ± 14% (*n* = 9) *inhibition* of current and a reversal potential of −44 ± 4 mV (*n* = 9) in FFA.

### AristA and TASK‐2 channels

**Figure 9:**
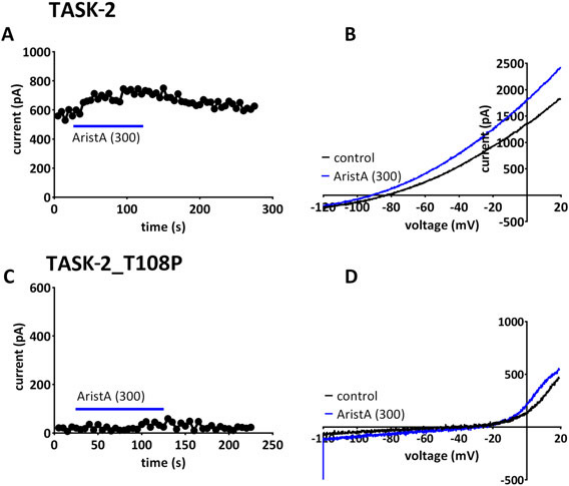
Regulation of WT TASK‐2 and TASK‐2_T108P mutant channels by aristolochic acid (AristA). (A) Representative time course for enhancement of outward current by AA (300 μM) for current through WT TASK‐2 channels. (B) Current–voltage relationships for WT TASK‐2 channels in the absence and presence of AristA (300 μM). (C) Representative time course for the outward current of TASK‐2_T108P channels in the presence and absence of AristA (300 μM). (D) Current–voltage relationships for TASK‐2_T108P channels in the absence and presence of AristA (300 μM).

At 100 μM, AristA has no effect on WT TASK‐2, TASK‐2_T108C or TASK‐2_T108P channels. However, at a concentration of 300 μM, AristA was able to induce a very modest enhancement of WT TASK‐2 channels (Figure 9A and B, 21 ± 13%, *n* = 5). At this high concentration, AristA also produced a very small enhancement of TASK‐2_T108P channels (Figure 9C and D, 12 ± 6%, *n* = 5) although current density of these channels remained unaltered compared with the absence of AristA at 2 ± 1 pA/pF (*n* = 5). Furthermore, the reversal potential of these currents through TASK‐2_T108P in the presence of AristA in 2.5 mM external [K^+^] was −40 ± 4 mV (*n* = 5), still inconsistent with the presence of a K^+^ selective current.

## Discussion

**Figure 10:**
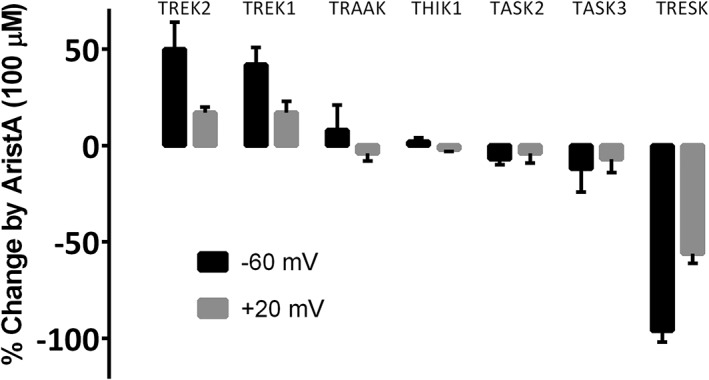
Effect of aristolochic acid (AristA) at 100 μM on representative human two‐pore‐domain potassium (K2P) channels. The effet on each channel is shown at two different voltages (−60 and +20 mV). Representative time courses for THIK‐1 and TASK‐3 channels are given in the Supporting Information.

In this study, we have shown that AristA has a range of actions on K2P channels. This is summarized for all K2P channels tested for a single concentration of AristA (100 μM) in Figure 10, where the percentage change in current in the presence of AristA compared with that in the absence of the compound is measured. The TREK family members, TREK‐1 and TREK‐2, are the only K2P channels to show substantive enhancement by AristA, whereas TRESK channels, which are the most distant from TREK channels in terms of sequence homology (e.g. Enyedi and Czirják, 2010; Feliciangeli *et al.*, 2015), are the only channels that are inhibited by AristA. There is no substantive effect of AristA, at this concentration, on TRAAK, TASK‐3, THIK‐1 or TASK‐2 channels.

### Mechanism of action of AristA

AristA is a non‐selective agent that has been suggested to regulate the activity of a number of different ion channels. For example, TRPA1−/− flies have been shown to have a reduced avoidance to AristA, and AristA induces action potential firing in these flies (Kim *et al.*, 2010). However, this effect of AristA is not linked to direct effect on TRPA1 channels (Kim *et al.*, 2010). AristA has also been demonstrated to influence the activity of certain K^+^ channels (Lopes *et al.*, 1998; Roch *et al.*, 2006), but again, these are supposed to be indirect actions. In these studies, AristA was proposed to work through block of the enzyme phospholipase A_2_ (Soderquist *et al.*, 2014). Phospholipase A_2_ is a calcium‐dependent enzyme that generates arachidonic acid. It is unlikely that block of phospholipase A_2_ underlies the substantive effects on TREK and TRESK channels seen here, because arachidonic acid enhances TREK (and TRAAK) channels (Fink *et al.*, 1996; Enyedi and Czirják, 2010) and blocks TRESK channels (Sano *et al.*, 2003; Kang *et al.*, 2004). Thus, AristA should produce the opposite effects to those observed here if it were working through block of phospholipase A_2_, namely, an inhibition of TREK (and TRAAK) channels and an enhancement of TRESK channels. Furthermore, we have shown that inhibition of TRESK does not depend on the intracellular calcium concentration determined by varying the concentration of intracellular calcium buffer, EGTA.

Inhibition of TRESK channels by AristA also does not work through calcium‐mediated stimulation of phosphatase activity, nor does it depend on the phosphorylation state of the channel on key serine residues in the intracellular loop between M2 and M3, known to be regulated by phosphatase activity (Czirják *et al.*, 2004; Czirják and Enyedi, 2006, 2010; Enyedi and Czirják, 2015), because it still caused substantive inhibition of both the permanently ‘phosphorylated’ form of the channel (S252E_S264E) and the permanently ‘dephosphorylated’ form of the channel (S252A_S264A). This can be contrasted with the block of TRESK channels by benzocaine, which *is* dependent on the phosphorylation state of these serine residues on the channel (Czirják and Enyedi, 2006).

Mutation of two bulky amino acids in the M2 and M4 inner pore regions of mTRESK channels (equivalent to F145A_F352A in hTRESK) has been shown to occlude the action of a number of different blocking drugs of the channel, and it was proposed that this position forms a binding site for blockers targeting TRESK channels (Kim *et al.*, 2013; Bruner *et al.*, 2014). These mutations also occlude the action of AristA and increase TRESK current density. Inhibition by AristA is voltage‐dependent, consistent with the idea that these amino acids, in the inner pore, may contribute to the binding site for AristA. However, we cannot exclude the possibility that these amino acids are important in regulating gating of the channel rather than forming a binding site, *per se*.

### Enhancement of TREK‐1 and TREK‐2 channels in the treatment of pain

TREK‐1−/− mice have been found to be more sensitive than WT mice to painful heat sensations (Alloui *et al.*, 2006). Furthermore, more recent work has shown that morphine, acting through μ opioid receptors, enhances TREK‐1 current directly. In the same study, TREK‐1−/− animals showed significantly less morphine‐induced analgesia than WT animals (Devilliers *et al.*, 2013). In both small‐ and medium‐sized dorsal root ganglion neurons, single‐channel and whole‐cell patch recordings suggested that TREK‐2 channels were most likely to underlie a substantial portion of background current present in these cells (Kang and Kim, 2006; Marsh *et al.*, 2012). More recently, TREK‐2 channels have been shown to be selectively expressed in IB4 binding C nociceptors (Acosta *et al.*, 2014) and contribute to the resting membrane potential of these neurons. As such, it was proposed that TREK‐2 expression might act to limit pathological pain (Acosta *et al.*, 2014). Enhancement of both TREK‐1 and TREK‐2 channel activity by AristA would contribute to a therapeutically useful effect of this compound in pain and may help to explain the persistent use of plant extracts containing this compound in herbal remedies for pain (see Introduction; IARC, 2002, 2012).

### Inhibition of TRESK channels and pain

The potential importance of TRESK channels in pain associated with migraine was highlighted by the observation that familial migraine with aura is associated with a dominant‐negative mutation in TRESK channels (Lafrenière *et al.*, 2010; Enyedi and Czirják, 2015). This suggests that decreased TRESK channel activity might exacerbate migraine pain and that activators of TRESK channels might provide a useful therapeutic approach in pain, particularly given their rather restricted expression in certain central neurons, spinal cord and nociceptive sensory neurons (Kang and Kim, 2006; Dobler *et al.*, 2007; Lafrenière *et al.*, 2010; Marsh *et al.*, 2012).

Hydroxy‐α‐sanshool, a primary active ingredient of Szechuan peppers, has been shown to block TRESK channels, and this action on TRESK channels has been proposed to underlie the distinctive numbing effect induced by this natural, widely used analgesic (Bautista *et al.*, 2008). This is a rather paradoxical observation given that TRESK channel activity is inhibited rather than enhanced by hydroxy‐α‐sanshool. However, it is possible that the tingling paraesthesia (Lennertz *et al.*, 2010) induced by hydroxy‐α‐sanshool eventually leads to desensitization of the excited neurons and a numbing of the sensation of pain in a manner analogous to that seen for the nociceptive agent, capsaicin, albeit through a different molecular mechanism (Mathie, 2010; Mathie and Veale, 2015). Because AristA is a potent inhibitor of TRESK channels, it might be predicted to act in a similar manner to hydroxy‐α‐sanshool to produce analgesia.

The voltage dependence of the effect of AristA on TREK‐1, TREK‐2 and TRESK channels shows that the effect of the compound is most prominent around the resting membrane potential of cells. This suggests that, in sensory neurons, AristA is most effective at membrane potentials that regulate the excitability and firing frequency of these neurons either dampening (for TREK‐1 and TREK‐2) or increasing (for TRESK) excitability. However, given that different K2P channels contribute differently to the resting membrane potential in different neuronal subpopulations, this is likely to be a complex, non‐linear mechanism.

### TASK‐2 channels and BEN

The mutation T108P in TASK‐2 channels is found in a number of patients diagnosed with BEN. This suggests that this mutation predisposes these patients to the disease because the proportion of BEN patients with this mutation is much larger than that seen in the general population (Toncheva *et al.*, 2014). We have found that this mutation markedly reduces TASK‐2 channel current density and alters the ion selectivity of the current, so that it is no longer potassium selective. Because we do not have a functional, tagged form of TASK‐2_T108P at present, we cannot exclude the possibility that this channel gives a small current because it does not express well at the membrane. A more conservative mutation at the same site (T108C) also reduces current density but not to nearly the same degree and slightly alters ion selectivity. This threonine residue is located at the top of the M2 transmembrane domain of TASK‐2, close to the channel selectivity filter, and it is present in 13 out of the 15 mammalian K2P channels (Mathie *et al.*, 2010). In other K2P channels, this threonine residue is known to influence channel gating (Zilberberg *et al.*, 2001; Mathie *et al.*, 2010). External alkalization (to pH 8.4) and application of FFA were shown to enhance current through both WT TASK‐2 channels and TASK‐2_T108C channels to around the same degree. These two treatments, however, caused no enhancement of TASK‐2_T108P channels and did not influence the ion selectivity of this mutated channel.

TASK‐2 has an important role in renal function (Cid *et al.*, 2013). Indeed, it is highly expressed in renal tissue and was originally isolated and cloned from human kidney (Reyes *et al.*, 1998). It is highly expressed in the basolateral membrane of proximal tubule cells (Warth *et al.*, 2004). TASK‐2−/− mice have been shown to have a reduced capacity for HCO_3_^−^ transport, which leads to proximal renal tubular acidosis (Warth *et al.*, 2004; Sepúlveda *et al.*, 2015). Poor or non‐functioning TASK‐2 channels in patients carrying the T108P mutation would be anticipated to produce similar effects in these patients and compromise renal function. Furthermore, because TASK‐2 channels form functional dimers, it is likely that the T108P mutation will act as a dominant negative in these patients.

TASK‐2 channels expressed in the retrotrapezoid nucleus regulate the ventilator response to CO_2_ (Bayliss *et al.*, 2015), but a parallel pathway mediated through the proton‐activated receptor GPR4 acts as a distinct central mediator of respiratory chemosensitivity (Kumar *et al.*, 2015). In this way, independent molecular pH sensors provide redundancy for this vital physiological function. It is possible, therefore, that similar independent parallel processes may exist to compensate for TASK‐2 in the kidney, if required.

We have found that AristA has little effect on either WT TASK‐2 channels or on TASK‐2_T108P channels except to induce a very modest enhancement of current through these channels at a high concentration (300 μM). This suggests that AristA is unlikely to interact directly with TASK‐2 channels in contributing to its action in BEN. Rather, loss of functional TASK‐2 channels in BEN (such as in patients with the T108P mutation) may indirectly increase susceptibility to AristA toxicity (and thus to BEN) acting through independent molecular pathway(s).

## Author contributions

A.M and E.L.V participated in research design, E.L.V conducted experiments, E.L.V and A.M performed data analysis, A.M and E.L.V wrote the manuscript.

## Conflict of interest

The authors declare no conflicts of interest.

## Declaration of transparency and scientific rigour

This Declaration acknowledges that this paper adheres to the principles for transparent reporting and scientific rigour of preclinical research recommended by funding agencies, publishers and other organizations engaged with supporting research.

## Supporting information


**Figure S1** Aristolochic acid (AristA) has no effect on the two pore domain potassium channels, TASK3 and THIK1
