# Supplementary material for: Aristolochic acid, a plant extract used in the treatment of pain and linked to Balkan endemic nephropathy, is a regulator of K2P channels
Source: Br J Pharmacol. 2016 Apr 5;173(10):1639–52. doi: 10.1111/bph.13465 (PMC4842925; doi:10.1111/bph.13465)
Supplement: Supplementary file 1 — Figure S1 Aristolochic acid (AristA) has no effect on the two pore domain potassium channels, TASK3 and THIK1 [file BPH-173-1639-s001.pdf]

Supplementary Figure

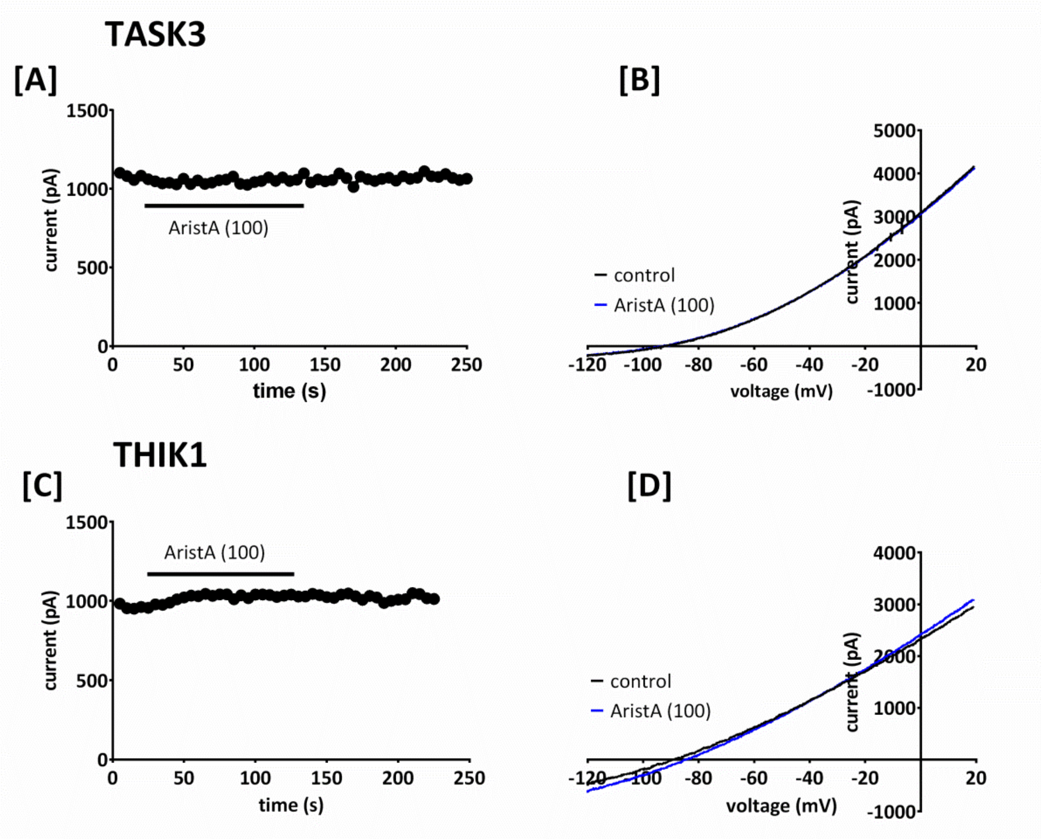

Aristolochic acid (AristA) has no effect on the two pore domain potassium channels, TASK3 and THIK1. (A) Time course plot showing lack of effect of AristA (100 µM) on human TASK3 Application of AristA is indicated by the bar. (B) TASK3 currents evoked by ramp changes in voltage in control conditions and in the presence of 100 µM AristA. (C) Time course plot showing lack of effect of AristA (100 µM) on human THIK1 (D) THIK1 currents evoked by ramp changes in voltage in control conditions and in the presence of 100 µM AristA.
